# Surface functionality and electrochemical investigations of a graphitic electrode as a candidate for alkaline energy conversion and storage devices

**DOI:** 10.1038/srep22056

**Published:** 2016-02-26

**Authors:** Ahmed B. Soliman, Hesham S. Abdel-Samad, Sayed S. Abdel Rehim, Hamdy H. Hassan

**Affiliations:** 1 Chemistry Department, Faculty of science, Ain-Shams University, Abbasia, Cairo, 11566, Egypt

## Abstract

Graphite is a typical electrocatalyst support in alkaline energy conversion and storage devices such as fuel cells, supercapacitores and lithium ion batteries. The electrochemical behaviour of a graphite electrode in 0.5 M NaOH was studied to elucidate its surface structure/electrochemical activity relationship. Graphite voltammograms are characterized by an anodic shoulder AI and a cathodic peak CI in addition to the oxygen reduction reaction plateaus, PI and PII. AI and CI were attributed to oxidation and reduction of some graphite surface function groups, respectively. Rotating ring disk electrode (RRDE) study revealed two different oxygen types assigned as inner and outer oxygen. The inner oxygen was reduced via the more efficient 4-electron pathway. The outer oxygen reduction proceeded with a lower efficient 2-electron pathway. The calculated percentages of the 4-electron pathway were ranged from 70% to 90%. A full mechanism for the graphite surface function groups changes over the studied potential window was suggested through the combination between the voltammetric, FT-IR and Raman results.

Fuel cells are considered to be perfect candidate as alternative energy resources. Yet the major technical problem facing their commercialization is basically due to using the high cost Pt based electrocatalysts^1^,^2^. Many researches focused on using Ni based electrocatalyst as a strong alternative to its Pt analogous^3^,^4^,^5^,^6^. During our research on electrodeposition of Ni electrocatalyst on graphitic support from different bathes to obtain more efficient and low cost fuel cell electrodes, we found that the electrochemical oxidation response obtained with the Ni electrodeposited from near neutral media is much higher than that obtained with Ni electrodeposited from sulphuric acid bath. At first glance, we attributed the low response of electrodes deposited from acidic media to smaller amounts of deposited Ni as a result of the preferential hydrogen evolution during its deposition. However, we have obtained a small response even for electrodes supported with Ni deposited from non aqueous methanol bath with null hydrogen evolution, Supplementary Fig. S1. Moreover, we have also observed a similar behaviour with Cu based electrocatalyst. Additionally, an increased activity of Pt for Methanol electrooxidation was reported if it would be supported on thoroughly oxidized glassy carbon, GC, owing to bi-functional catalysis^7^,^8^. All these observations derived us to think about the presence of synergic effect between the graphitic support and the electrodeposited active phase. These remarks were the motive beyond our passion to stands on the true role of graphitic supports.

Graphite is considered as one of the most widely used electrodes in different electrochemical applications either as anode^9^,^10^,^11^,^12^,^13^ or cathode^14^. Many of these applications neglected the complicated surface nature of graphite^15^,^16^ and the possible interactions between the surface functional groups (SFG’s) and the solution species and/or their reaction products^15^. The electrochemical behaviour of the graphite electrode is strongly dependent on the possible organic surface compounds combinations of carbon and oxygen^15^,^16^.

One of the most recent applications of the graphite electrode in direct alkaline fuel cell is to act as a large surface support for the electro-catalytic phase^4^,^17^,^18^. Almost all studies dealt with the graphite electrode surface as a non-interacting support and tackled it from surface area point of view^4^. In this work, it has proved that the SFG’s undergo critical changes within the medium and the potential window in which the graphite electrode play a vital indirect role in both electrochemically obtained behaviour and response.

Carbon surfaces are very susceptible for both oxygen chemisorption and physisorption^19^,^20^. The origin of the SFG’s in the graphite is attributed mainly to the chemisorbed oxygen^15^. They are composed of alcoholic, phenolic, carboxylic, lactonic, oxygen containing alicyclic compounds, … etc.^15^,^16^. Obviously, it must be taken into consideration that these functional groups may undergo chemical and/or electrochemical transformations depending on the electrolyte and the potential at which they are subjected^21^.

Y. Chabal and his co-workers^22^ utilized the FT-IR spectroscopy to follow the effect of thermal reduction on the SFG of graphene oxide. However, it is well known that the ordinary quantitative analysis using FT-IR is restricted by several limitations^23^.Although Raman and IR spectroscopy are complementary techniques widely used to provide useful information about graphitic surface functional groups^24^, many researchers investigated the Raman D and G bands for graphite, graphene oxide and graphene, yet they only considered the I_D_/I_G_ for testing the quality of the produced graphene^25^,^26^,^27^ neglecting the correlation between the I_D_/I_G_ ratio with the surface functionality of the graphitic surfaces. The assignment of the peaks of the G-bands reported in the literature is not straightforward. Few studies correlated the overlapped peaks of the G-band for nano diamond (ND) to the C=C, G-band, OH and C=O surface functional groups^24^,^28^.

The aim of this work is to investigate the major significant role of the graphite SFG’s and their effects on using the graphite as a support for the electrocatalytic active phase in direct alkaline fuel cell.

## Results and Discussion

### Cyclovoltammetric behavior of BG in alkaline media

The studies in the field of enhancing the performance of graphite based fuel cell electrodes need a prior inspection of the surface structure of graphite and its relationship with the electrochemical response of bare graphite electrode (BG). The study of the cyclic voltammetric behaviour of bare graphite electrode in alkaline media is a mandatory for understanding the processes that happen on its surface.

**Figure 1:**
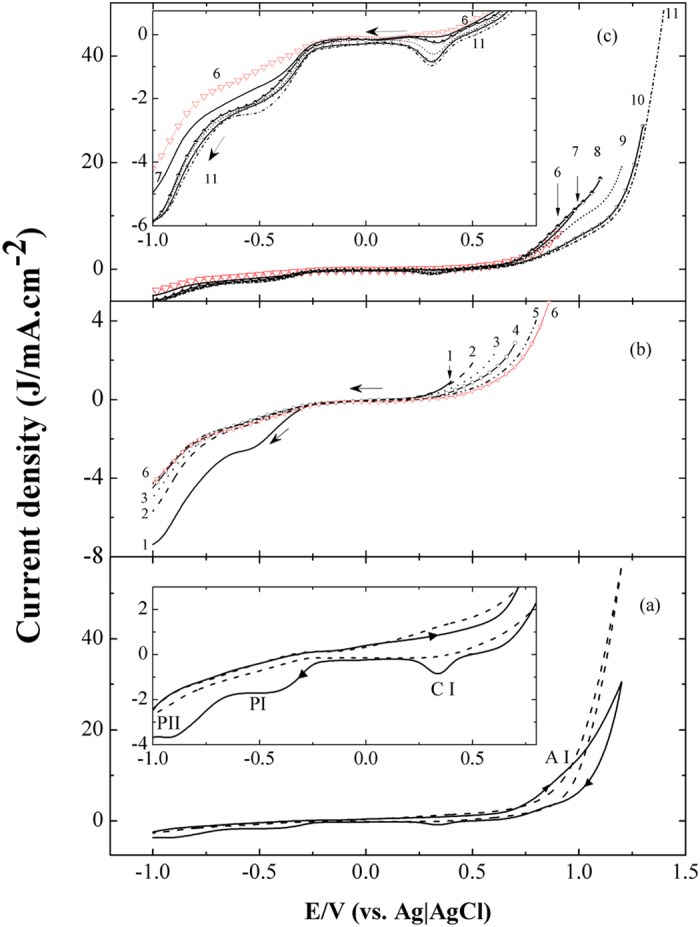
(a) The CV response of the bare graphite (BG) electrode at 50 mV.s^−1^ in 0.5 M NaOH without (solid line) and with 1M EtOH (dashed line). (b,c) are its LSV in 0.5M NaOH from various anodic potentials to −1 V. The inserts are zooming up of the nobler portion of the curves.

The cyclic voltammograms CV of bare graphite electrode in 0.5 M NaOH in absence and presence of 1M ethanol as fuel are represented in Fig. 1a. In the forward span, the observed cathodic current is usually attributed to the reduction of H_2_O and/or dissolved O_2_. The stationary electrode condition of Fig. 1 doesn’t favour the sluggish diffusion controlled oxygen reduction reaction, ORR. Therefore, water reduction reaction is predominant in this potential range. The anodic part of the forward span prior to E = 0.7 V does not exhibit well-defined peaks. However, beyond this potential the current increases with the anodic potential in two successive regions with different slopes. The first region covers the potential range till 1V and implies a small anodic peak assigned as AI; followed by a steeper sloped region at higher anodic potentials. Although the peak AI appears as a shoulder in Fig. 1a, it becomes well defined at lower scan rates, Supplementary Fig. S2.

In the reverse span, in addition to the two sloped regions, a cathodic peak CI is observed at 0.35 V. As an attempt to stand on the CI origin, a series of linear sweep voltammograms (LSVs) was carried out, Fig. 1(b,c). They commenced with different starting potentials, E_s_, more anodic than CI and ended with a constant final cathodic potential of −1 V. The first LSV started from 0.4 V and swept cathodically to −1V (curve 1), then the electrode potential was jumped to 0.5 V starting the second ramp (curve 2) without withdrawing the electrode from the solution. This procedure was repeated sequentially by shifting the start potential 0.1 V each time until arriving the final ramp (curve 11) which started from 1.4 and ended at −1 V. This set of experiments is divided into two groups, Fig. 1(b,c). It is worth noting that the cathodic peak CI does not appear in curves 1–7, where E_s_ varies from 0.4 to 1 V. However, when E_s_ exceeds E_AI_, CI appears and its height increases progressively as E_s_ moves to 1.4 V. It is clear that, CI is a result of the reduction of certain species formed in the potential range more anodic than AI where the increase in its height is synchronized with the shift in the E_s_ from 1.1 to 1.4 V, curves 8–11. To the best of our knowledge, CI is not reported in the published graphite voltammograms which lied in narrower anodic potential windows. However, Stevanovi**ć** *et al*.^8^ obtained a reversible peak around 400 mV (vs. SCE) during the electroactivation / anodization of glassy carbon, GC, in acidic solution. They related the redox peaks to the pseudocapacitive currents associated with the charge transfer of carbon or graphite surface functional groups (SFG`s). Since their peaks were enhanced upon intensification of the oxidative conditions, they concluded that electrochemical activation of carbon causes abundant functionalization of its surface and simultaneously opens the closed-pore structure of GC. The broadness of this peak was attributed to its composition of several overlapping peaks, as a result of the close redox responses of the functional groups of different types. Moreover, a cathodic peak in the potential range of CI was previously observed in the first sweep during the electrochemical grafting of GC in 0.1 M KOH^29^.

On the other hand, at potentials more cathodic than CI in the voltammogram of Fig. 1a, the reverse scan exhibits two well defined cathodic plateaus PI and PII. The height of these plateaus enhances with increasing the potential scan rate, Supplementary Fig. S2. Similar plateaus were previously obtained for flat metallic electrodes only under hydrodynamic conditions (using RDE) which enhance the diffusion controlled oxygen reduction reaction responsible for PI and PII^30^,^31^. The presence of such plateaus in the voltammograms of stationary GE reveals the presence of high amount of captured oxygen in the surface GE vicinity.

Moreover, the LSVs illustrated in Fig. 1(b,c), show the two plateaus in curve 1, but they diminish in curves (2–6), with a starting potential lies just before the range of AI. Subsequently, the two plateaus return to the LSVs whose starting potentials equal to or exceed that of AI, Fig. 1c. It is clear that the currents of the two plateaus don’t alter when E_s_ exceeds E_AI_, (E_s_ > 1V), where the GE surface becomes fully saturated with oxygen.

The above results reveal that the reduction process associated with the cathodic peak CI and the two plateaus PI and PII are independent from each other. This observation arises from the appearance of PI and PII without the existence of CI (curves 1 and 7) and the increase of the CI current without a similar response on PI and PII (curves 8–11). Moreover, we can conclude that PI and PII are correlated to a reduction of inherent (captured) oxygen species attached to the surface that produced in the potential range of the anodic peak AI, rather than the naturally dissolved O_2_ in the electrolyte. This inherent oxygen can be subdivided into two types of oxygen; oxygen in the form of oxidized graphite functional groups (OxSFG) and adsorbed or trapped oxygen. The oxidation of the different SFG commences with the easily oxidizable groups, E-OxSFG, in the potential range of AI and proceeds though the more anodic potential till oxidizing the most difficult ones, D-OxSFG. Consequently, during the reverse scan the D-OxSFG is reduced easily at CI. On the other hand, PI and PII could be due to the reduction of the adsorbed or trapped oxygen. A more detailed studies are proposed for the deeper inspection of the oxygen reduction reaction using hydrodynamic techniques (RDE, RRDE) as well as, an intensive inspection of FT-IR spectra of GE’s subjected to different electrochemical conditions.

### ORR and RDE studies

**Figure 2:**
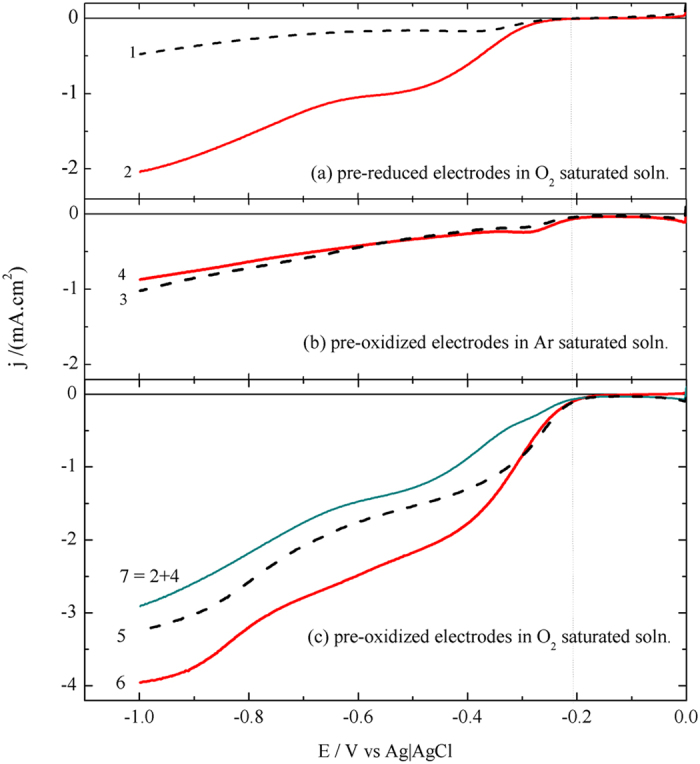
LSV of a pre-oxidized at 1.3V (b,c) and pre-reduced at −1 V (a) graphite disc electrodes for 60 s in Ar saturated (b) and O_2_ saturated (a,c) 0.5 M NaOH at 5mV.s^−1^. Black dashed lines and red solid lines denote stationary and 1600 rpm rotating electrode, respectively.

The two plateaus PI and PII were observed in the literatures under hydrodynamic conditions and assigned to oxygen reduction reaction (ORR). Some of the reported works^32^,^33^,^34^,^35^,^36^ proposed a reduction mechanism via two successive two-electron processes (equation 1, 2, 3) or direct 4-electron mechanism (equation 4)^35^,^36^. However, others attributed the occurrence of the two plateaus to the change of surface active sites such as quinine-like groups that are produced at highly anodic potential^37^. The aim of this part of the present work is to correlate the plateaus currents and the types of oxygen to be reduced. For this purpose we ascribed some experiments whose results are shown in Fig. 2. The idea is to differentiate between dissolved (outer) O_2_ that diffuses to the electrode surface from the bulk of solution, and the inherent (inner) oxygen, OxSFG and the adsorbed oxygen that are formed at the graphite surface and within its pores as a result of oxidation at highly anodic potentials. Figure 2a shows LSV curves for graphite electrodes pre-reduced at −1V to get ride off any inner O_2_ that may previously present on the electrode surface. The sluggish diffusion controlled outer O_2_ reduction enhanced when the electrode rotates at 1600 rpm as expected^30^,^31^. When the electrode is exposed to pre-oxidation at 1.3V it forms a sufficient amount of inner oxygen as will be illustrated in the following sections. The curves of Fig. 2b show that reduction of the inner oxygen is not significantly affected by the convection or rotation of the electrode where it is already present in the electrode vicinities. It is also clear from this figure that the activity of ORR is enhanced significantly as its onset potential of the ORR shifts to more positive values after pre-oxidation (compare curves of Fig. 2a,b). Figure 2c illustrates the reduction of both of inner and outer oxygen. It is worth noting that the currents corresponding to the reduction of inner and outer oxygen (curve 6) are larger than the algebraic sum of curves 2 (outer O_2_) and 4 (inner oxygen), Fig. 2. These results prove that the pre-oxidation at 1.3 V is not only saturates the pores of the graphite surface, but also creates some new or modifies active sites for ORR and hence enhances the reduction of both of inner and outer oxygen. This suggestion is confirmed by the positive shift of the onset potential of the ORR after pre-oxidation, Fig. 2, and by earlier work of Palitero *et al*.^32^.

### RRDE measurements

**Figure 3:**
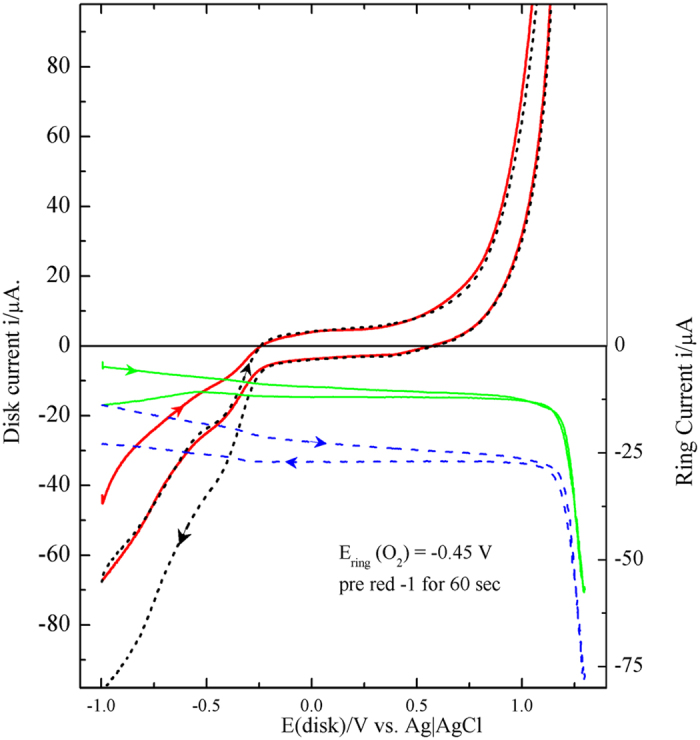
RRDE experiments with a BG disk response pre-reduced at −1.0 V for 60 s in Ar deaerated 0.5 M NaOH at 5 mV.s^−1^ and rotated at 1600 rpm. The Pt ring potential was fixed at − 0.45 V for O_2_ detection. Solid and dashed lines denote first and second cycles, respectively.

**Figure 4:**
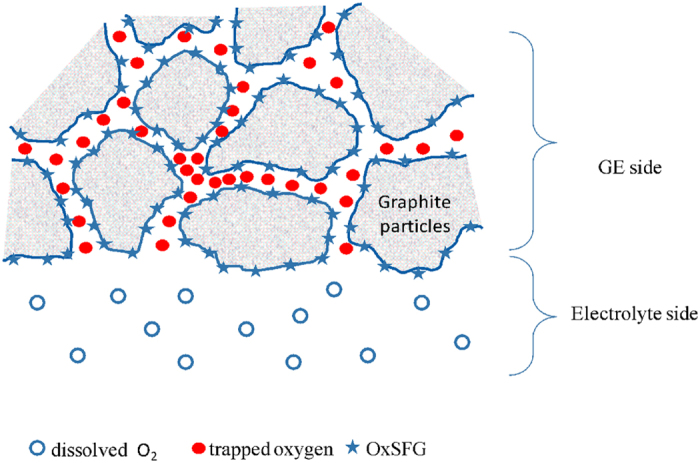
Graphical representation of the distribution of different types of oxygen on the graphite electrode surface.

One step forward on the road of identifying the different processes that happen on the BGE surface during the different potential regions of its CV is the rotating ring disk electrode (RRDE) study. Figure 3 shows the result of RRDE experiment designed to detect the evolution of oxygen through two successive disk CV’s by fixing the ring potential at the experimentally selected proper ORR potential at Pt surface (E_ring_ = −0.45V). Prior to this experiment, the disc GE is reduced by keeping its potential at −1 V for 60 s to reduce all of the inner oxygen species present in the vicinity of the electrode surface and deactivate the ORR active sites. The ring current during the forward span of the first disc cycle exhibits low background values in the potential range −1.0 to 1.0 V. More anodic disc potentials show high rate of O_2_ evolution which is detected at the ring causing high increase in their cathodic currents at 1.1 V > E_disk_ > 1.3 V. The same trend is observed in the second cycle (dashed line). It is obvious that the background ring current (base line) increases continuously with time as reflected by its successive increase through the four ring spans. These results are evidences that the produced O_2_ in the potential range 1.1 V > E_disk_ > 1.3 V is able to do the following: adsorption on the GE, trapping inside the graphitic pores and oxidation of SFG (inner oxygen). In addition, the remaining of the produced O_2_ dissolves in the electrolyte (outer O_2_). Figure 4 is a graphical representation of the distribution of the different types of oxygen on the graphite surface. The disc rotation repels the inner O_2_ to the ring and enhances diffusion of the outer dissolved O_2_ towards the ring through the entire disc CV. The slight increase of the background ring current (base line) is a result of accumulation of the produced O_2_ through the successive spans.

The oxygen reduction reaction (ORR) on graphite surfaces in alkaline media was demonstrated to follow a complex reaction scheme^38^. The most important feature in this scheme is that it proceeds via one of the following two over-all pathways^32^,^39^: the first, the less efficient^35^,^36^, is a two successive 2–electron steps pathway that involves peroxide intermediate;


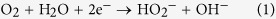


followed by


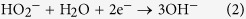


or the decomposition (disproportionation) reaction^39^,


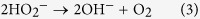


Earlier ^18^O experiments showed the transfer of O_2_ into HO_2_^−^ as a unit without bond rupture^40^.

The second pathway, the more efficient, is a direct 4 electron mechanism^35^,^36^:


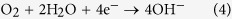


**Figure 5:**
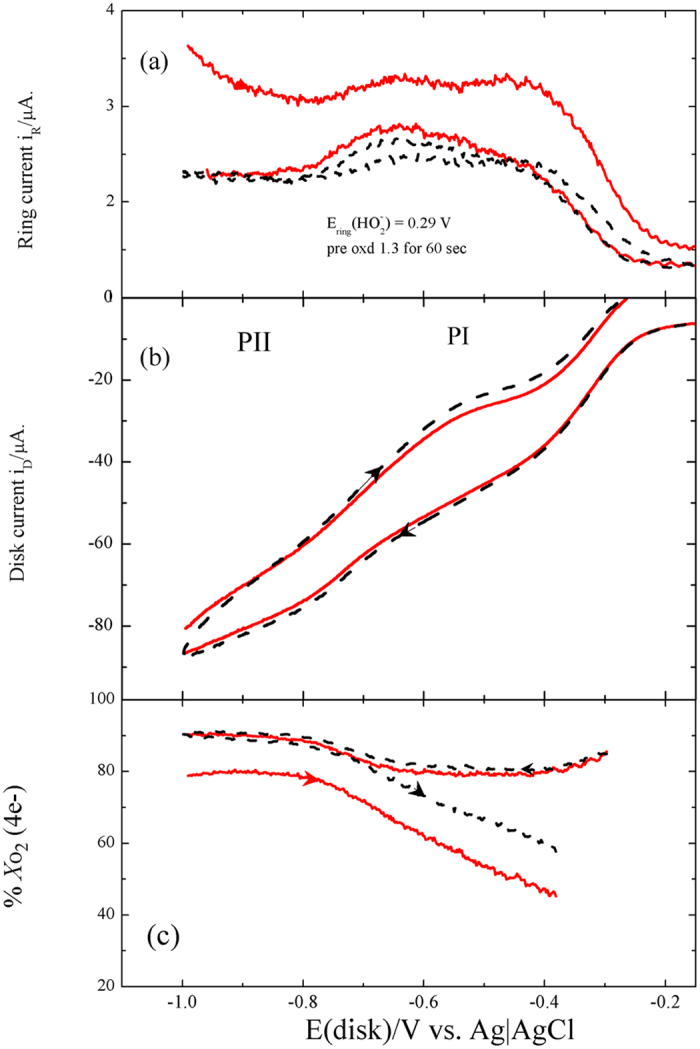
RRDE experiments with a BG disk response (**b**) pre-oxidized at 1.3V for 60 s in naturally aerated 0.5 M NaOH swept from −1 to 1.3 V at 5 mV.s^−1^ and rotated with 1600 rpm. The Pt ring potential was fixed at 0.29 V for HOO^−^detection (a). Dashed black and solid red lines denote first and second cycles, respectively. (c) is the calculated percentage of O_2_ reduction current proceeds via 4-e pathway % 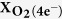 calculated on the bases of eq. (5, 6, 7, 8) using the data of (a,b).

The RRDE experiment of Fig. 5 was carried out in a naturally aerated 0.5 M NaOH for a disc pre-oxidized at 1.3 V for 60 s. The ring potential was fixed at 0.29 V to detect the 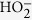 species produced at the disc rotating at 1600 rpm^38^. The disc potential was swept from −1 to 1.3 V in a naturally aerated 0.5 M NaOH. The ring oxidation currents of Fig. 5a prove the 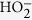 production in the potential range of PI, however, in the potential range of PII the amount of 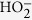 decreases. These results confirm the occasion of ORR via the peroxide pathway (equations 1, 2) and support the possibility of its decomposition reaction (equation 3). This suggestion is supported by the slight increase in the O_2_ detection currents in the potential range of PI, PII, Fig. 5. The fraction of O_2_ (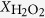) that is reduced via the 2e peroxide pathway was calculated using equations (5, 6, 7) which considers disk current, *I*_D_, as the sum of the O_2_ reduction currents to water (4e), 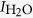, and to H_2_O_2_ (2e), 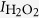 (equation 5)^38^. The collection efficiency *N* was experimentally determined and found to be 0.151:


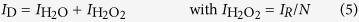



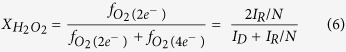


where 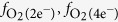 are the molar flux rate of H_2_O_2_ and O_2_, respectively. They were calculated according to equation (7):


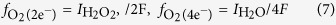


On the bases of these equations, Fig. 5c shows a relation between the disc potential and the calculated (% 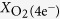) percentage of O_2_ reduced through the 4-e pathway of equation (4), where:


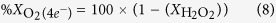


Although, the earlier work of Yeager and his co-workers^39^ nominated graphite among the surfaces on which O_2_ reduction proceeds in alkaline media through peroxide pathway, a recent work^36^ demonstrated values more than 3.6 for the number of the electron transferred per O_2_ molecule in the potential range of the less cathodic plateau PI. Figure 5a shows that 90%, 80% of O_2_ is reduced along the more efficient 4-electron pathway in the potential ranges of PI and PII, respectively. The low values of current densities for HO_2_^−^ ring oxidation currents were previously explained in terms of the strong interaction with the graphite functional groups during its reduction^39^. It may also be attributed to the increase of the concentration of paramagnetic centres during the anodic oxidation of graphite due to the formation of aryloxy radical which is stabilized by the delocalization of the unpaired electrons in the π- system of carbon^32^.

### FTIR measurements

Deep inspection of Fig. 1 provokes many questions which deserve answers; for what extent the SFG may play role in the oxidation and reduction processes that occurred at the surface of GE; how the different oxygen species that reduced at PI and PII are linked to the surface of graphite; finally, what is the nature of the different SFG formed or changed at different potential regions?

**Figure 6:**
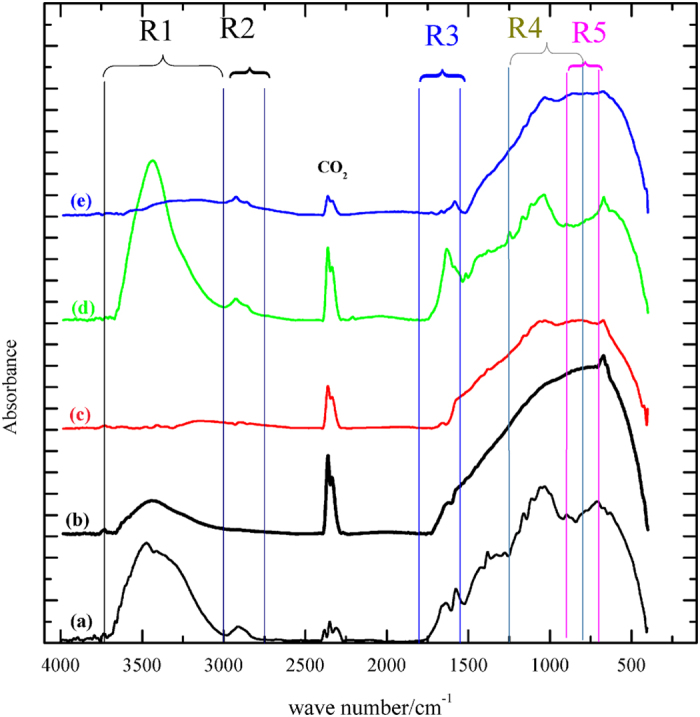
FT-IR spectra of BG as is (a) and after immersion in 0.5 M NaOH for 10 min. (b), then oxidized at 1.3 V for 10 min. (c) after that, it is reduced at the potential of CI (0.35V) (d). The spectrum of a new BG after its direct reduction at −1.1V in a new solution for 10 min. is (e).

To get answers for these questions an extensive FT-IR study has been made for a GE treated in different manners, Fig. 6, as follows: spectrum (a) was obtained from a blank bare graphite electrode BGE (as is), and dipped in 0.5 M NaOH for 10 min., spectrum (b). In addition, the GE was subjected to oxidation in 0.5 M NaOH at 1.30V for 10 min. (spectrum (c)), followed by reduction at 0.35 V (the potential corresponds to CI) for 5 min. (spectrum (d)). Moreover, spectrum (e) belongs to a new GE exposed directly to reduction at −1.1V for 10 min.

From the first glance, the FT-IR spectra are very complicated especially in the overlapped finger print region (600–1500 cm^−1^). This overlap is similar to that observed in the FT-IR of thermally reduced graphene oxide investigated by Chapal and his co-workers^22^. To resolve the peaks of their spectroscopic data using cluster-based first principle calculations, they identified many oxygenated functional groups in the fingerprint region. The overlapping in the fingerprint region results from the complicated surface structure of the graphite due to the effect of the compatible neighbouring groups coupling such as; carboxyl-carboxyl, hydroxyl-hydroxyl, carbonyl-hydroxyl and any cross coupling between them. If coupling between carbonyl and hydroxyl group occurs, it could be quite observable. This can be attributed to the strong coupling arising from the similarity in the bond lengths of these groups (1.20 å and 1.23 å, respectively)^22^.

**Figure 7:**
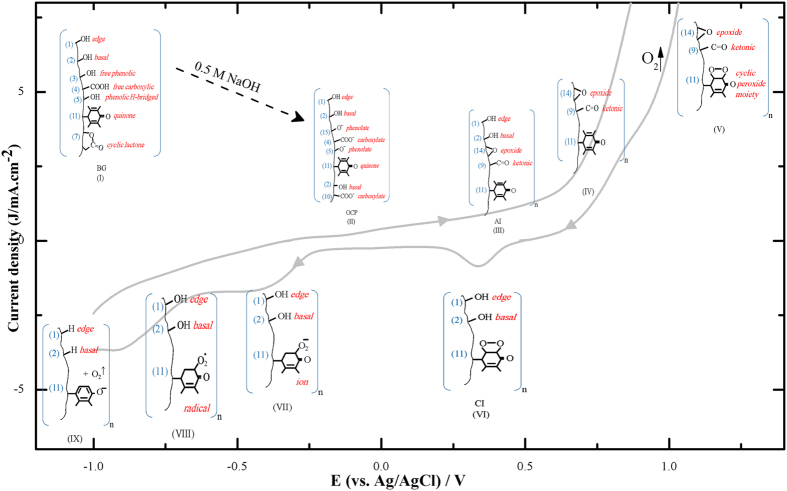
A suggested mechanism correlated to the different potential regions of the cyclic voltammogram of Fig. 1.

The assessment of the FT-IR spectra raises wide range of the SFG distribution possibilities. Some of them were excluded based on the comparison of the FT-IR spectra of Fig. 6 side by side with the conclusions derived from the above results. A list of suggested SFG possibilities is given in Table (1). In addition, a proposed mechanism for the various processes occurred along the studied potential range is demonstrated in Fig. 7.

The major oxygen containing functional groups on the GE surface demonstrated in FT-IR spectra of Fig. 6 are divided into five spectral regions, R1-R5. The spectrum of the BGE is characterized by the marked appearance of different O-H’s bands (R1 & R2); a variety of functional groups containing olefinic C=C and carbonyl C=O bands (R3); epoxide and C-O stretching (R4) and phenolate or mono substituted phenyl groups (R5) with a small CO_2_ band which occurs at about 2300 cm^−1^, Fig. 6a and Table 1 (and references therein). This gives the possibility to raise a proposed structure (I) for the BG, Fig. 7. Upon immersing the GE in 0.5 M NaOH, the O-H’s bands (R1 & R2) diminish greatly with enhancing the CO_2_ band without a noticed change in the C=O band, Fig. 6b. This is mainly attributed to the disappearance of all acidic OH groups due to their conversion into phenolate and carboxylate anions. The residual low intensity peak observed at (R1) is attributed to a small number of unchanged alcoholic based O-H’s, which may exist at the edge and basal sites as illustrated in structure (II).

Furthermore, the O-H’s and the carboxylic C=O bands vanish after oxidation at 1.30 V, Fig. 6c and structures (III–V) of Fig. 7. On the contrary, they reappear again after post reduction at 0.35 V, leaving the spectra nearly the same as the blank, spectrum (a), Fig. 6d and structures (VI–VIII). Finally, reduction at −1 V gives rise to the disappearance of the O-H’s, CO_2_ and the carboxylic C=O bands, spectrum (e), structure (IX). This result is in a good agreement with the observed diminution of the two plateaus PI and PII, Fig. 1b (curves 1–7) upon pre-reduction. All of the above mentioned structures were based on selected possibilities of function group interchanging^21^ under either electro-oxidation or electro-reduction^41^.

In addition, two step oxidations of the SFG’s are suggested at the anodic potentials of AI and above. The first step is the oxidation of the surface carboxylates and phenolates (E-OxSFG) forming epoxide and ketonic surface C=O in addition to generating extra quinone sites added to the already existed ones at AI potential (structure II → III). In the second step, at higher anodic potentials, the edge and the basal surface OH groups (D-OxSFG) are oxidized (structure (IV)). Moreover, oxygen gas is evolved and stored on the surface by the highly oxygen susceptible quinone sites forming cyclic peroxide/quinone moiety^15^,^42^, as shown in structure (V) of Fig. 7. This conclusion clarifies the way of the above mentioned modification of the graphite surface active sites to accommodate the more efficient 4-electron ORR pathway as discussed above. On mild reduction at 0.35 V, some of O-H’s returns again to the surface and is manifested by the appearance of CI. However, the peer reduction at −1 V leads to the removal of the C-OH and carboxylic C=O groups from the surface, which is reflected by the disappearance of the OH bands (R1 & R2) and the vanishing of quinonidal R3 bands (structure (IX)). Structures (VII) and (VIII) are introduced adopting the well known previously suggested mechanism for ORR at graphitic surfaces^15^,^39^,^42^.

### Raman measurements

**Figure 8:**
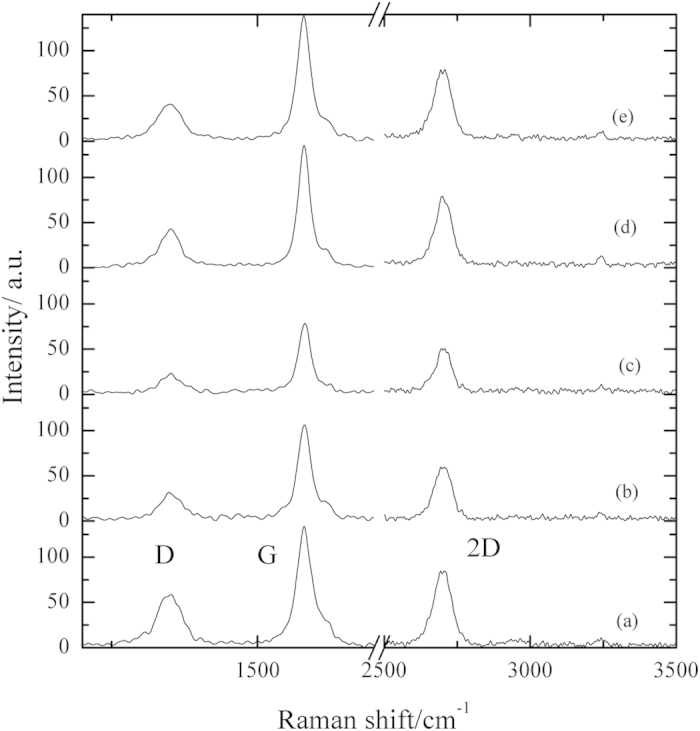
Raman spectra of graphite samples, the notations: (a–e) have the same descriptions illustrated in Fig. 6.

**Figure 9:**
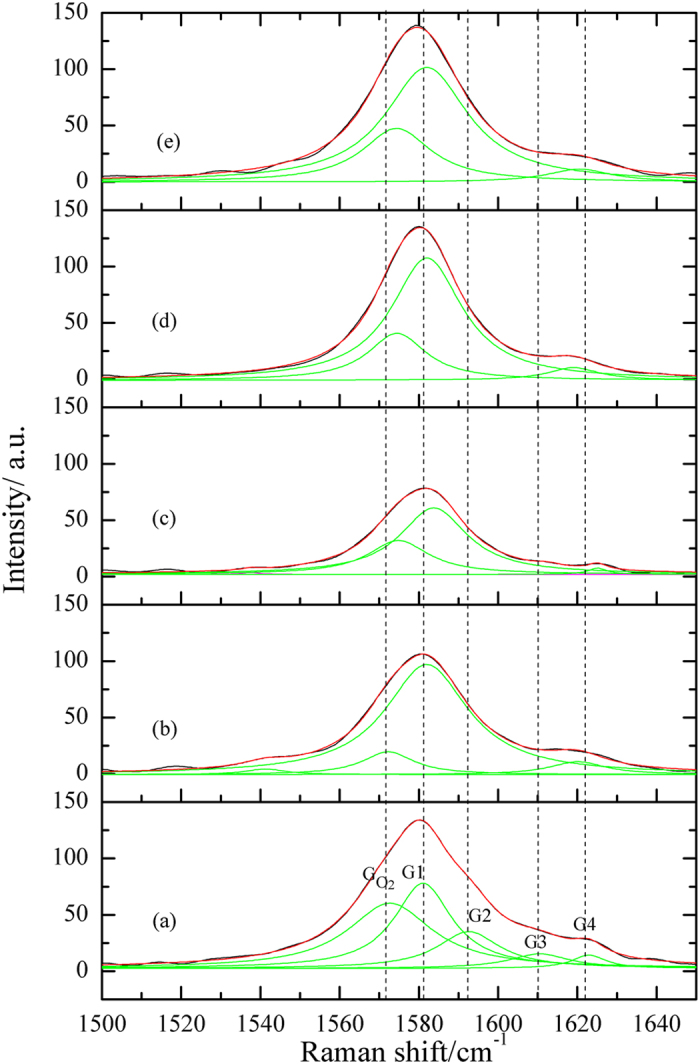
Multi-peak fitting of G-bands of Raman spectra of graphite samples, the notations: (a–e) have the same descriptions illustrated in Fig. 6.

In order to confirm the FT-IR results, Raman spectroscopic study was performed. Figure 8 represents typical Raman spectra for the graphite samples (a–e) as previously described in the FT-IR study, Fig. 6(a–e). They exhibit the characteristic Raman features of graphitic carbon; the G-band at ~1582 cm^−1^, the disorder-induced D-band at ~1350 cm^−1^ and the 2D-band at 2700 cm^−1^. Deep inspection of the asymmetric shape of the broad G-band indicates its convolution of multi overlapping peaks. Figure 9 represents the deconvolution of their graphite G-band using the Lorentzian multi peak fitting. It shows the existence of five sub peaks allocated around 1572 cm^−1^ (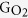), 1581 cm^−1^ (G1), 1592 cm^−1^ (G2), 1610 cm^−1^ (G3) and 1623 cm^−1^ (G4).

For the first glance, it is clear that G1 present in all Raman spectra. It may represent the famous characteristic sp^2^/sp^3^ carbon structure peak of graphite. We believe that it is not worthy to use the absolute heights or areas of the Raman bands in their arbitrary units scale to quantify their changes through different spectra. For this reason we normalize the G’s peak areas with respect to the area of their own G1, the results are tabulated in Table 2. The presence of G2 in the BG surface, Fig. 9a, and its disappearance in the subsequent curves strongly confirms its attribution to the acidic –OH SFG’s. Similar behaviour was obtained for G3 and probably correlated to the hydrolysis of lactonic ring present on the BG surface by dipping in alkaline media. These results are in accordance with the transformation of stucture (I) to stuctures (II–IX) as suggested in the mechanism of Fig. 7.

It is worthy note that the deconvolution process is not able to separate the band to its all individual constituents. Some resulted peaks such as G4 may compose of overlapping Raman signals of C=O stretching and hydroxylic OH bending. The change of the contribution of the hydroxylic OH’s and C=O’s signals to G4 varies with the treatment conditions^24^,^43^. The %G4 remains nearly constant before and after dipping in NaOH solution, where the hydroxylic OH’s and C=O are involved in the transformation of structure (I) to structure (II) except the conversion of carboxylic acid into carboxylate, Fig. 7. However, the %G4 decreases greatly on oxidation because of the hydroxylic OH’s oxidation, the decarboxylation process and the removal of CO_2_ (structure II → V). Moreover, the %G4 re-increases again in reduction at 0.35 V due to the ketonic and epoxide SFG’s reduction (structure V → VI). Further reduction leads to slight increase of C=O character as a result of cyclic per-oxo-quinone reduction which raises the aromatic character (structure VI → IX), Fig. 7.

On the other hand, 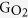 is assigned to the well known Raman O_2_ band^44^,^45^. For the BG sample, %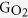 is high due to the atmospheric O_2_ enclosed in the graphite pores in addition to the adsorbed O_2_. Upon dipping in NaOH followed by drying with Ar stream, the %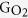 decreases to ~13% of G1 as a result of O_2_ dissolution in the solution and the occupation of the graphite pores by Ar atoms which has no Raman signals. Moreover, at high anodic potential (1.3 V) O_2_ evolution reaction occurs in parallel to the oxidation of the GSFG’s as previously discussed in RRDE results (Fig. 3). Consequently, %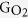 increases to about ~46% of G1. Furthermore, shifting the potential cathodically ceases O_2_ evolution reaction which diminish %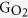 again.

## Conclusion

In this work, we report a novel insight on the structure/activity relationship of graphite electrode (GE) in alkaline medium and a special concern was subjected towards the ORR on its surface. Our findings strongly confirm that the surface function groups (SFG) of the graphite could be divided into easily and more difficult oxidized groups, E-OxSFGs and D-OxSFGs, respectively. The E-OxSFGs are mainly assigned to carboxylates, and phenolates, while the D-OxSFGs are mainly assigned to the edge and the basal hydroxyl sites. A novel cathodic peak CI is recorded at 0.35 V in the cyclic voltammograms of GE in alkaline medium and attributed to the reduction of the D-OxSFGs. Moreover, at high anodic potentials, sufficient amount of oxygen is produced at the GE surface and divided into inner (inherent) and outer oxygen. The inherent oxygen is captured into the graphite surface in the form of oxidized graphite functional groups (OxSFG) in addition to the adsorbed or trapped O_2_ within the graphite pores. The excess of the produced oxygen is dissolved in the electrolyte (outer O_2_). The appearance of PI and PII plateaus in stagnant conditions could be ascribed to the reduction of the adsorbed or trapped oxygen rather than the dissolved outer O_2_. The inner (inherent) oxygen reduces mainly through the more efficient 4-electron pathway while the outer oxygen reduces mostly via a lower efficient 2- electron pathway. On the bases of the FT-IR and Raman spectroscopy results, a full mechanism with nine proposed structures, is suggested to understand the possible surface functional groups interchanges of the GE during its electrochemical polarization specially ORR. We believe that this suggested mechanism will add a great contribution for maximizing the efficiency of the different forms of electrochemical energy conversion and storage devices utilizing graphite as electrocatalyst support through tuning of the functionality of the graphitic support surface.

## Methods

All solutions were prepared from analytical grade chemicals and double distilled water. All experiments were carried out in 0.5 M NaOH solutions in a jacketed EuroCell kit provided by Gamry instruments, USA. The working electrode was a Graphite electrode GE (G0091) provided by EG&G (USA) with an apparent geometrical surface area of 0.28 cm^2^. The counter electrode was a platinum wire electrode. All of the potentials were recorded against saturated double junction Ag|AgCl reference electrode. Polishing of the working electrode with emery papers of 280 to 1000 grades was done until a smooth electrode surface is obtained, followed by rinsing with distilled water prior to each experiment. A fresh solution and newly polished electrode is used with each experiment. All measurements were maintained at room temperature (25 ± 1 °C). The measurements were done just after connecting the three electrodes and immersing the working electrode in the electrolyte.

All electrochemical measurements were done using a reference 3000 and interface 1000 Gamry electrochemical workstations provided with a Pine instrument rotating disk and rotating ring disk electrodes, RDE, RRDE, respectively. FTIR spectroscopic analysis of the electrode surfaces was done using Thermo Nicolet 6700 spectrometer. Raman spectra were recorded using Brucker Sinterra energy dispersive Raman. Excitation was carried out using 532 nm Laser beam with a power of approximately 10 mW.

## Additional Information

**How to cite this article**: Soliman, A. B. *et al*. Surface functionality and electrochemical investigations of a graphitic electrode as a candidate for alkaline energy conversion and storage devices. *Sci. Rep*. **6**, 22056; doi: 10.1038/srep22056 (2016).

## Supplementary Material

Supplementary Information

## Figures and Tables

**Table 1:**
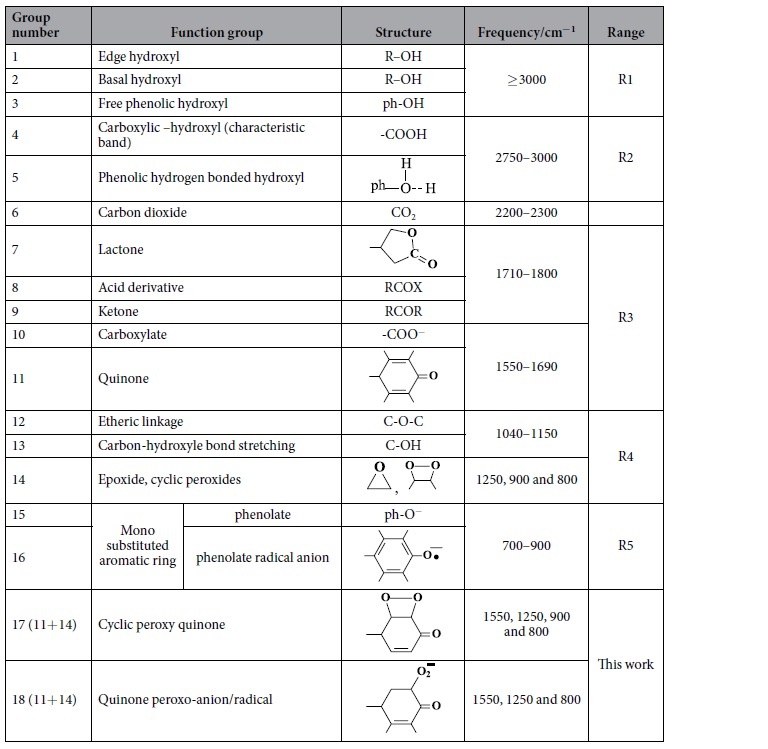
The FT-IR spectroscopic data of the suggested possible graphite SFG^22^,^23^.

**Table 2:** The Raman spectroscopic data of the deconvoluted peaks of the G-bands of the different graphite samples of Fig. 9.

Peak symbol	Peak position ±2 cm^−1^	%Gn =(Gn/G1)x100					Peak assigned to
		(a)^*^	(b)^*^	(c)^*^	(d)^*^	(e)^*^	
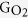	1572	112.4	12.9	46.2	31.4	39.0	O_2_
G1	1582	100	100	100	100	100	sp^2^/sp^3^ carbon character
G2	1592	47.0	–	–	–	–	OH acidic
G3	1610	17.9	–	–	–	–	C=O lactonic
G4	1622	9.3	8.0	2.7	7.6	8.8	C=O + OH hydroxylic

^*^The notations: (a–e) have the same descriptions illustrated in Fig. 6.
