# Supplementary figures and images for: Surface functionality and electrochemical investigations of a graphitic electrode as a candidate for alkaline energy conversion and storage devices
Source: Sci Rep. 2016 Feb 26;6:22056. doi: 10.1038/srep22056 (PMC4768093; doi:10.1038/srep22056)

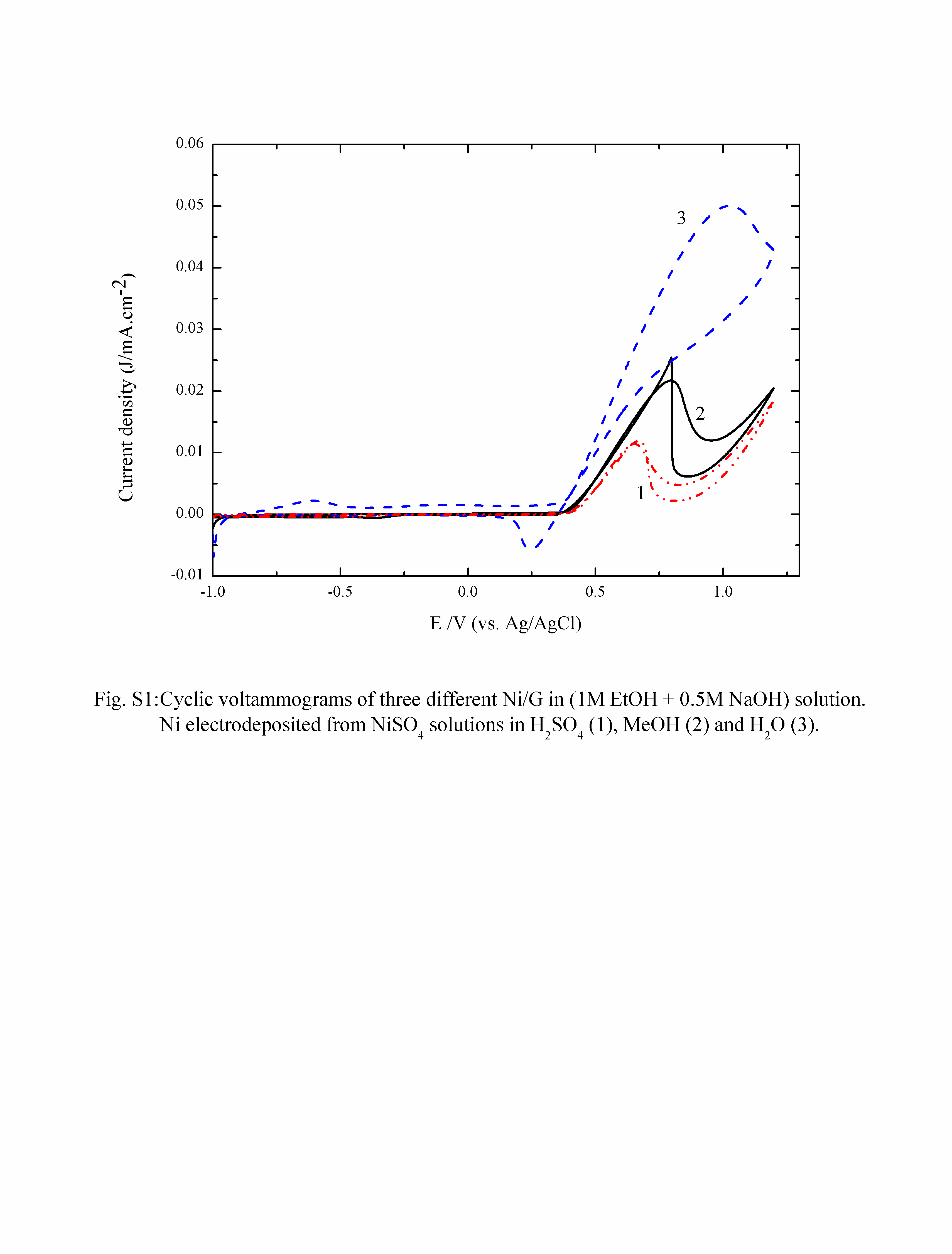


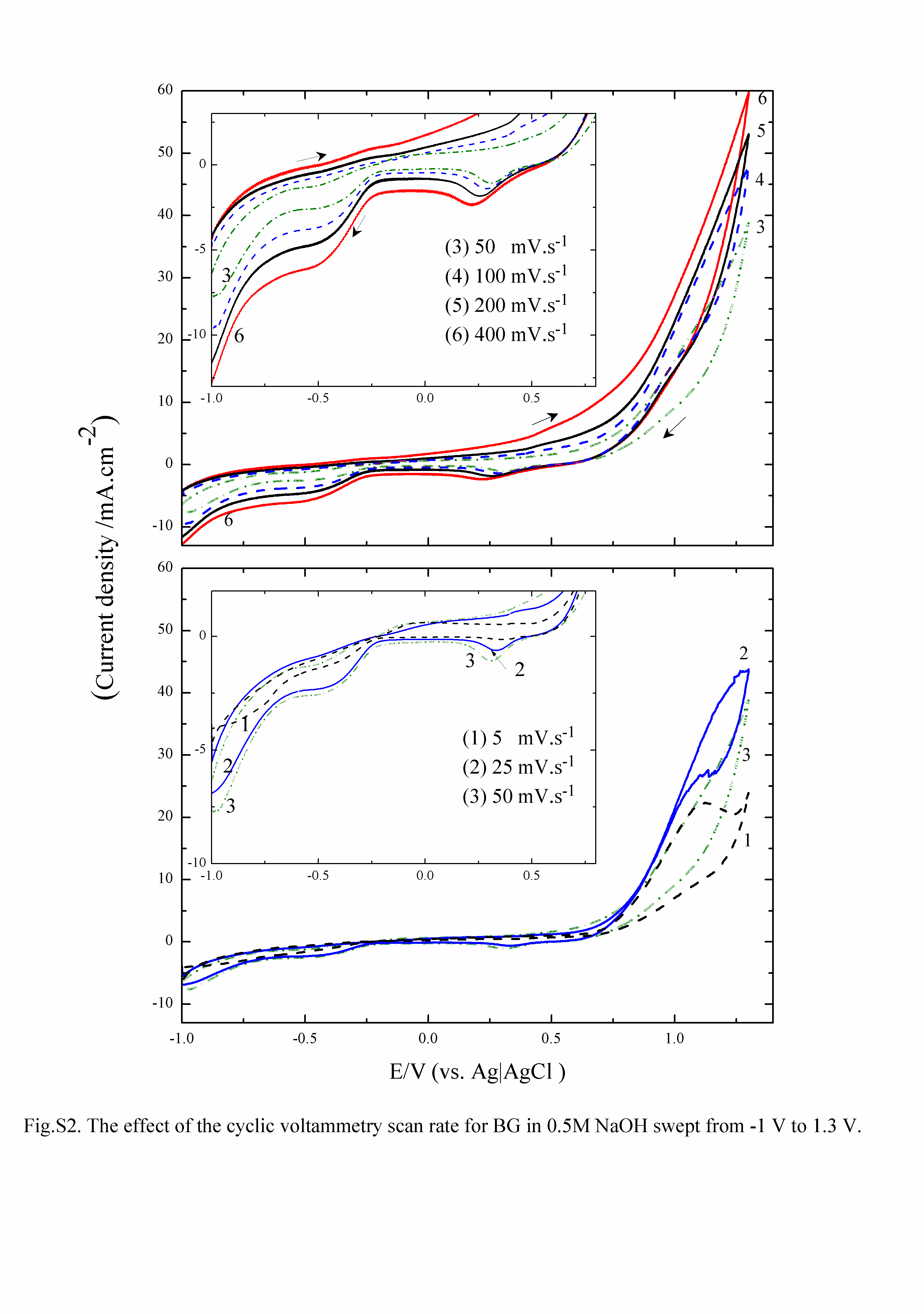

Supplement: Supplementary Information [file srep22056-s1.doc]
